# Characterization of Striatal Neuronal Loss and Atrophy in the R6/2 Mouse Model of Huntington’s Disease 

**DOI:** 10.1371/currents.hd.48727b68b39b82d5fe350f753984bcf9

**Published:** 2014-01-06

**Authors:** Lindsay Dodds, Jianfang Chen, Kiersten Berggren, Jonathan Fox

**Affiliations:** Department of Veterinary Sciences, University of Wyoming, Laramie, Wyoming, USA; Department of Veterinary Sciences, University of Wyoming, Laramie, Wyoming, USA; Department of Veterinary Sciences, University of Wyoming, Laramie, Wyoming, USA; Department of Veterinary Sciences, University of Wyoming, Laramie, Wyoming, USA

## Abstract

Striatal neuronal degeneration and loss is an important feature of human Huntington’s disease (HD). R6/2 HD mice recapitulate many features of human HD including striatal atrophy. While striatal neuronal atrophy and loss is reported in R6/2 HD mice the degree of neuronal loss and the characteristics of cell body atrophy are unclear. We used stereological approaches to estimate whole striatal neuronal numbers and characterize changes in striatal neuronal size distribution. R6/2 HD mice had ~126000 fewer neurons per striatum (~12% decline) at 12 weeks of age than wild-type litter-mates; differences were not present at 5 weeks. Analysis of striatal neuronal numbers per cell body size category revealed declines in neuron numbers in the size ranges 550-1050 µm3 suggesting that larger striatal neurons are more susceptible to atrophy or loss in late stages of disease. R6/2 HD mice have a striatal neuronal loss phenotype. As striatal neuronal loss in human HD is dramatic, neuronal loss in R6/2 striatum provides an important late-stage outcome measure for study of disease modifying interventions

## Introduction

Human Huntington’s disease (HD) is characterized by slowly progressive brain atrophy primarily affecting striatum and cerebral cortex 1 ****that develops in the pre-clinical phase of the disease process 2 . There is considerable evidence that atrophy of striatum results from a combination of neuronal degeneration and loss. Vonsattel et al 1 reported loss of 50% of caudate neurons in neuropathology grade I and 95% in grade IV human disease. Neurons within the striatum comprise ~95% medium spiny neurons which degenerate early in HD and remaining interneurons which are less susceptible to degeneration.

Numerous genetic mouse models of HD exist and these have proven invaluable for studying disease mechanisms and therapies 3 . R6/2 mice express exon 1 of the human huntingtin gene containing an expanded CAG repeat 4 . These mice experience rapid disease progression with symptoms beginning around 6 weeks of age, large behavioral declines by 12 weeks 5 , and death as early as 10 weeks of age in some colonies (e.g. 6 ). R6/2 mice recapitulate many features of human HD including progressive brain atrophy involving neostriatum and cerebral cortex, weight loss, motor dysfunction and accumulation of mutant huntingtin aggregates in brain neurons. While these mice express only a fragment of human mutant huntingtin evidence has been provided that this fragment occurs endogenously in mouse HD 7 .

YAC128 HD mice express full-length human mutant huntingtin from a yeast artificial chromosome. They have a slowly progressive HD-like disease process. Slow 8 and colleagues reported a 15% decrease in striatal volume at 9 months of age, and a 15-18% reduction in striatal neuronal estimates at 12 months of age in these mice. Knock-in models of HD also exhibit late-onset neurodegeneration. For example, in the CAG140 mouse HD model 38% and 40% reductions in striatal volume and striatal neuron estimates are reported, respectively, in 20-26-month-old animals 9 . While fragment mouse models of HD (e.g. R6/2) are genetically less accurate than full-length models their rapid disease progression and HD-like phenotype has resulted in their continued use.

Several approaches have been used to demonstrate striatal neuronal degeneration in HD models. R6/2 HD mice have increased activity of neuronal caspase 1 and 3 and changes in Bcl-2 family members supportive of activation of striatal neuronal apoptosis 10 . One study reported significant mitochondrial abnormalities in neurons of R6/2 mice 11 . Findings from TUNEL labeling for DNA nicks have however been variable 11 ^,^ 12 . Several studies have demonstrated the presence of neuronal cell body atrophy in the striatum of R6/2 HD mice 13 ^,^ 14 . However, we are aware of only one study that has estimated striatal neuronal numbers in R6/2 mice as a means to quantify neurodegeneration. Stack et al 14 estimated neuronal numbers in the anterior 2/3 of the striatum, to the level of the anterior commissure, and found significantly fewer neurons at 12-weeks of age in R6/2 mice compared to wild-type littermates. The purpose of the current study was to complete a detailed stereologic analysis of the entire striatum, in particular characterizing neuronal numbers and size changes with disease progression.

## Methods

All procedures were pre-approved by the University of Wyoming Animal Care and Use Committee (IACUC). R6/2 HD mice were bred and maintained as previously described 5 . CAG repeat sizes were determined from genomic DNA by Laragen Inc. (Culver City, CA) and averaged 181 with a standard deviation of 6. Mice were sacrificed by deep anesthesia then intra-cardiac perfusion with 4% paraformaldehyde and processed for stereology as described 15 . In brief, brains were sectioned coronally to include the entire striatum at 40 µm thickness; every 12^th^ section was mounted and stained using the thionin method. We used the unbiased optical dissector method to estimate striatal neuronal numbers 16 . We used a counting frame size of 40 x 40 µm and a grid size of 500 x 500 µm. During the optical dissector procedure we also estimated striatal neuronal cell body volume using the 2D nucleator with five isotropic uniform random sections per cell. All slides were coded so the operator was unaware of the genotype. The numbers of mice per group were: wild-type 5-weeks n=15; HD 5-weeks n=13; wild-type 12 weeks n=9; and HD 12 weeks n=10. The average number of neurons / mouse analyzed was 384 for 5-week mice (range: 245-521) and 359 for 12-week mice (range: 272-436). For each mouse, neurons were binned into 50 µm^3^ cell body size categories using Microsoft excel software. The estimated total number of striatal neurons in each size category in wild-type and HD mice was then analyzed using the students t-test in SAS software version 9.2 (Cary, NC, USA).

## Results

R6/2 HD mice had 12.7% fewer striatal neurons at 12-weeks of age (p=0.035) (**Fig. 1A**). Wild-type mice had a mean of 993891 ± 38483 (SEM) neurons; R6/2 HD mice had a mean of 868043 ± 38483 (SEM). To determine if this difference was due to neuronal loss or developmental effects we studied 5-week-old mice. As shown in **Fig. 1B** there were no differences at 5-weeks of age indicating the presence of neuronal loss in R6/2 mouse striatum occurring between 5 and 12 weeks. Use of the nucleator stereologic tool has been reported in numerous studies to characterize changes in striatal neuronal cell body volume in HD mice; however, distribution of neuronal sizes and number estimates by cell size were not determined (e.g. [12,13]). We analyzed neuronal population estimates on a cell body size basis (**Fig. 2**). At 12 weeks, the most significant finding was a loss of neurons in the size range 550-1050 µm^3 ^(**Fig. 2A**). Within smaller neuron size categories (200-500 µm^3^) neuronal population mean estimates were increased in R6/2 mice but this did not approach significance (**Fig. 2A**). At 5 weeks, there were no significant differences in neuron number estimates per size category (**Fig. 2B**).

While there is abundant biochemical and morphologic evidence to support neuronal degeneration in R6/2 mouse striatum 10 ^,^ 11 ^,^ 17 , we are not aware of reports of morphologic evidence of late-stage neuronal death events in R6/2 striatal neurons. Our findings, and those of others, suggest that while the process of neuronal degeneration is gradual, neuronal death and removal is rapid. We estimated the number of striatal neurons in the process of death when sacrificed at 12-weeks of age. To do this we assumed that neuron death in R6/2 mice begins at 60 days of age 14 and that the process is linear across time (a constant number of neurons dying / day). Based on this, we calculated that R6/2 HD mice lose ~6909 neurons / day / striatum. In one rodent model of acute neuronal injury cell death was detected ~6 hours post-insult 18 . In one study phagocytosis of apoptotic neurons occurred within 1.5 hours of introduction of microglia into cultures 19 . Based on these findings we assumed that in R6/2 mice the process of morphologically detectable neuronal death and removal lasts 8 hours. From this we calculated that a 20 µm section of striatum taken at the level of the anterior commissure of a 12 week R6/2 mouse would contain ~4 dying neurons. Therefore, based on our estimates, the kinetics of neuronal degeneration, loss and removal underlies the difficulty of identifying neuronal death morphologically. Stereologic approaches to quantifying neurons, as used here, can overcome these difficulties.

**Striatal neuronal loss occurs in R6/2 HD mice:**
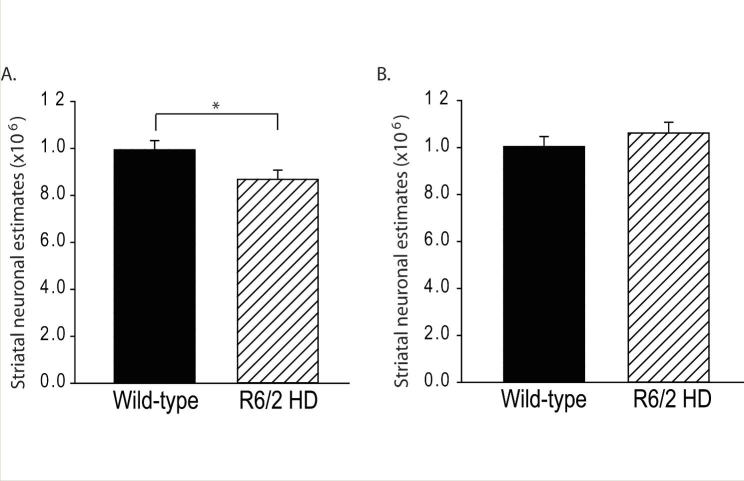
Striatal neuronal loss occurs in R6/2 HD mice. **A.** R6/2 HD mice have 12.7% fewer neurons than wild-type litter-mate mice at 12 weeks of age. p=0.035, **B.** R6/2 HD mice do no differ from wild-type litter-mate mice in striatal neuronal number estimates at 5 weeks of age. P-value: *=p<0.05 ****

**Striatal neuronal atrophy and loss in R6/2 HD mice.**
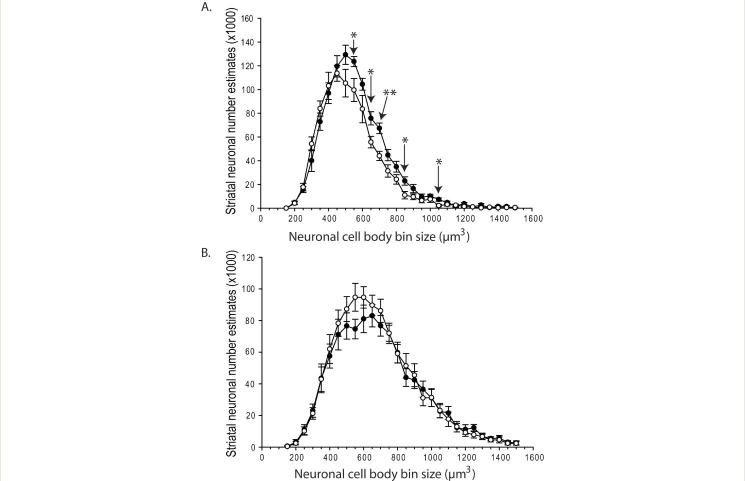
Striatal neuronal atrophy and loss in R6/2 HD mice. **A.** R6/2 HD mice have fewer neurons in the cell body size range 550-1050 µm^3 ^at 12 weeks of age. **B. **There are no significant differences in neuronal number estimates per cell body size category in 5-week-old mice. Round circles = R6/2 HD; closed circles=wild-type. P-values: *=p<0.05, **=p<0.01

## Discussion

Numerous studies have documented a reduction in striatal neuronal cell body size in R6/2 HD mice 13 ^,^ 20 . Here we took a new approach to analysis by determining estimates of neuronal numbers in specific size categories. Our analysis cannot clearly reveal in what population neuronal loss occurs in R6/2 striatum as decreased numbers of neurons in the size range 550-1050 µm^3^ (**Fig. 2A**) could result from atrophy, loss or a combination. However, the results do suggest that larger medium spiny neurons are more vulnerable to degeneration in R6/2 HD.

Taken together, our findings corroborate and extend those reported previously 14 . Our analysis of striatal neuron number within whole striatum reveals a 12% loss of neurons in R6/2 mice which is less than the 26% loss reported in an analysis based on examination of the anterior striatum to the level of the anterior commissure, which is about 2/3 of the striatum 14 . This discrepancy could be for a number of reasons that includes differences in neuronal loss in whole striatum versus anterior striatum and differences in the respective R6/2 mouse models including CAG size and background genetic effects due to the backcrossing approach to colony maintenance. Importantly, however, all mouse models of HD studied so far have significantly less striatal neuronal loss than the 90% reported in advanced human HD 1 . While striatal neuronal loss in R6/2 mice and other mouse HD models is modest, the presence of dramatic loss in human HD brain suggests that estimation of striatum neuronal number should be an important outcome in HD mice which can be used to evaluate therapies in pre-clinical studies. Finally, we undertook a sample size analysis to determine the number of mice required to detect rescue of neuronal loss in R6/2 mice in a hypothetical treatment study. Parameters used were: 80% power, a significance level of 0.05, standard deviations proportional to the mean and a one-tailed t-test 21 . Based on these parameters we calculated that for 50% and 100% rescue of striatal neuronal loss it would be necessary to have group sizes of 10 and 5, respectively. While stereologic approaches to estimate neuronal loss can be labor intensive, our calculations indicate that only modest group sizes are required. Analysis of neuronal numbers may be especially valuable for interventions that are protective in mice and when further characterization of protective effects is needed prior to considering human testing.

## References

[1] Vonsattel JP, Myers RH, Stevens TJ, Ferrante RJ, Bird ED, et al. (1985) Neuropathological classification of Huntington's disease. J Neuropathol Exp Neurol 44: 559-577. 10.1097/00005072-198511000-00003 2932539

[2] Tabrizi SJ, Scahill RI, Durr A, Roos RA, Leavitt BR, et al. (2011) Biological and clinical changes in premanifest and early stage Huntington's disease in the TRACK-HD study: the 12-month longitudinal analysis. Lancet Neurol 10: 31-42. 10.1016/S1474-4422(10)70276-3 21130037

[3] Ferrante RJ (2009) Mouse models of Huntington's disease and methodological considerations for therapeutic trials. Biochim Biophys Acta 1792: 506-520. 10.1016/j.bbadis.2009.04.001 PMC2693467 19362590

[4] Mangiarini L, Sathasivam K, Seller M, Cozens B, Harper A, et al. (1996) Exon 1 of the HD gene with an expanded CAG repeat is sufficient to cause a progressive neurological phenotype in transgenic mice. Cell 87: 493-506 10.1016/s0092-8674(00)81369-0 8898202

[5] Chen J, Marks E, Lai B, Zhang Z, Duce JA, et al. (2013 ) Iron Accumulates in Huntington’s Disease Neurons: Protection by Deferoxamine. PLoS ONE 8 10.1371/journal.pone.0077023 PMC3795666 24146952

[6] Chopra V, Quinti L, Kim J, Vollor L, Narayanan KL, et al. (2012) The sirtuin 2 inhibitor AK-7 is neuroprotective in Huntington's disease mouse models. Cell Rep 2: 1492-1497. 10.1016/j.celrep.2012.11.001 PMC3534897 23200855

[7] Landles C, Sathasivam K, Weiss A, Woodman B, Moffitt H, et al. (2010) Proteolysis of mutant huntingtin produces an exon 1 fragment that accumulates as an aggregated protein in neuronal nuclei in Huntington disease. J Biol Chem 285: 8808-8823. 10.1074/jbc.M109.075028 PMC2838303 20086007

[8] Slow EJ, van Raamsdonk J, Rogers D, Coleman SH, Graham RK, et al. (2003) Selective striatal neuronal loss in a YAC128 mouse model of Huntington disease. Hum Mol Genet 12: 1555-1567. 10.1093/hmg/ddg169 12812983

[9] Hickey MA, Kosmalska A, Enayati J, Cohen R, Zeitlin S, et al. (2008) Extensive early motor and non-motor behavioral deficits are followed by striatal neuronal loss in knock-in Huntington's disease mice. Neuroscience 157: 280-295. 10.1016/j.neuroscience.2008.08.041 PMC2665298 18805465

[10] Zhang Y, Ona VO, Li M, Drozda M, Dubois-Dauphin M, et al. (2003) Sequential activation of individual caspases, and of alterations in Bcl-2 proapoptotic signals in a mouse model of Huntington's disease. J Neurochem 87: 1184-1192. 10.1046/j.1471-4159.2003.02105.x 14622098

[11] Yu ZX, Li SH, Evans J, Pillarisetti A, Li H, et al. (2003) Mutant huntingtin causes context-dependent neurodegeneration in mice with Huntington's disease. J Neurosci 23: 2193-2202. 10.1523/JNEUROSCI.23-06-02193.2003 PMC6742008 12657678

[12] Keene CD, Rodrigues CM, Eich T, Chhabra MS, Steer CJ, et al. (2002) Tauroursodeoxycholic acid, a bile acid, is neuroprotective in a transgenic animal model of Huntington's disease. Proc Natl Acad Sci U S A 99: 10671-10676. 10.1073/pnas.162362299 PMC125009 12149470

[13] Chopra V, Fox JH, Lieberman G, Dorsey K, Matson W, et al. (2007) A small-molecule therapeutic lead for Huntington's disease: preclinical pharmacology and efficacy of C2-8 in the R6/2 transgenic mouse. Proc Natl Acad Sci U S A 104: 16685-16689. 10.1073/pnas.0707842104 PMC2034257 17925440

[14] Stack EC, Kubilus JK, Smith K, Cormier K, Del Signore SJ, et al. (2005) Chronology of behavioral symptoms and neuropathological sequela in R6/2 Huntington's disease transgenic mice. J Comp Neurol 490: 354-370. 10.1002/cne.20680 16127709

[15] Fox JH, Connor T, Chopra V, Dorsey K, Kama JA, et al. (2010) The mTOR kinase inhibitor Everolimus decreases S6 kinase phosphorylation but fails to reduce mutant huntingtin levels in brain and is not neuroprotective in the R6/2 mouse model of Huntington's disease. Mol Neurodegener 5: 26. 10.1186/1750-1326-5-26 PMC2908080 20569486

[16] West MJ, Slomianka L, Gundersen HJ (1991) Unbiased stereological estimation of the total number of neurons in thesubdivisions of the rat hippocampus using the optical fractionator. Anat Rec 231: 482-497. 10.1002/ar.1092310411 1793176

[17] Turmaine M, Raza A, Mahal A, Mangiarini L, Bates GP, et al. (2000) Nonapoptotic neurodegeneration in a transgenic mouse model of Huntington's disease. Proc Natl Acad Sci U S A 97: 8093-8097. 10.1073/pnas.110078997 PMC16675 10869421

[18] Wang CZ, Johnson KM (2007) The role of caspase-3 activation in phencyclidine-induced neuronal death in postnatal rats. Neuropsychopharmacology 32: 1178-1194. 10.1038/sj.npp.1301202 16985504

[19] Witting A, Muller P, Herrmann A, Kettenmann H, Nolte C (2000) Phagocytic clearance of apoptotic neurons by Microglia/Brain macrophages in vitro: involvement of lectin-, integrin-, and phosphatidylserine-mediated recognition. J Neurochem 75: 1060-1070. 10.1046/j.1471-4159.2000.0751060.x 10936187

[20] Ferrante RJ, Kubilus JK, Lee J, Ryu H, Beesen A, et al. (2003) Histone deacetylase inhibition by sodium butyrate chemotherapy ameliorates the neurodegenerative phenotype in Huntington's disease mice. J Neurosci 23: 9418-9427. 10.1523/JNEUROSCI.23-28-09418.2003 PMC6740577 14561870

[21] Owen DB (1965) The Power of Student's t-test. Journal of the American Statistical Association 60: 320-333.

